# Clinical, surgical, and endocrine outcome following treatment of posterior pituitary tumors: a retrospective cohort study

**DOI:** 10.1007/s11102-025-01518-z

**Published:** 2025-04-05

**Authors:** Natalia Kremenevski, Oliver Schnell, Roland Coras, Michael Buchfelder, Nirjhar Hore

**Affiliations:** 1 Department of Neurosurgery, Friedrich-Alexander Universität Erlangen-Nuremberg, Erlangen, Germany; 2 Department of Neuropathology, Friedrich-Alexander Universität Erlangen-Nuremberg, Erlangen, Germany

**Keywords:** Pituicytoma, Granular cell tumor, Spindle cell oncocytoma, Pituitary function, Endocrine and surgical outcomes

## Abstract

**Purpose:**

This study evaluates the clinical presentation, endocrine dysfunction, surgical outcome, and long-term prognosis in patients with histologically confirmed posterior pituitary tumors (PPTs).

**Methods:**

A retrospective cohort study was conducted on 19 patients treated for PPTs at a single center between 2000 and 2023. Data on clinical, endocrine, and surgical outcomes were collected and analyzed.

**Results:**

The cohort included 3 pituicytomas (PCs), 8 granular cell tumors (GCTs), and 8 spindle cell oncocytomas (SCOs) patients, with a female predominance (58%) and a mean age of 57.2 ± 13.2 years. Symptoms leading to diagnosis were headache (31.6%), visual impairment (21%), and sexual dysfunction (10.5%). GCT patients had higher preoperative BMI (34.49 ± 5.72) compared to PC (22.12 ± 2.40) and SCO (24.74 ± 4.24) patients (*p* < 0.01). Postoperative BMI increased across all groups, with GCTs patients showing the steepest rise (*p* < 0.01). Endocrine dysfunction largely persisted or worsened after surgery, with limited recovery at follow-up. Surgical approaches included transsphenoidal (48%) and transcranial (52%), achieving gross total resection in 58% of cases. Tumor recurrence occurred in 16% of patients, all requiring adjuvant radiation therapy. Tumor-specific survival at 5 years was 100% with an overall survival rate of 80% where non-tumor-related comorbidities accounted for the observed mortality.

**Conclusion:**

PPTs are rare tumors with significant endocrine and metabolic consequences. While surgical management is associated with favorable tumor-specific survival, persistent endocrine dysfunction and postoperative progressive BMI underscore the need for long-term follow-up and targeted interventions. These findings contribute to the understanding of PPT biology and support the development of optimized management strategies.

## Introduction

Recent advancements in neuroendocrinology and oncology have led to increased attention to the rare entity of posterior pituitary tumors (PPTs), with additional impetus stemming from the updated WHO classification anchored in molecular and histological criteria - particularly in terms of recognition of a common single-cell origin for neurohypophyseal tumors [1–4]. PTTs encompass three main subtypes: pituicytomas (PCs), granular cell tumors (GCTs), and spindle cell oncocytomas (SCOs) [4, 5].

Largely due to their rarity, the epidemiology and natural history of PPTs remain poorly understood despite recent progress in research. These tumors account for less than 1% of all pituitary neoplasms, with PCs, SCOs, and GCTs representing approximately 0.1%, 0.4%, and 0.5% of all sellar masses respectively. Existing data primarily derives from small case series and meta-analyses, which are subject to selection biases and hampered by inconsistent reporting [5–11].

PPTs are low-grade, non-neuroendocrine neoplasms characterized by slow growth and clinically nonfunctional behavior [6–8, 10]. Mass effect on surrounding parasellar structures constitutes the primary reason behind development of symptoms, manifesting as visual disturbances (acuity and/or visual field), headaches, or endocrine dysfunction [11–13]. However, clinical, radiologic, and biochemical features of PPTs are nonspecific, frequently mimicking more common pituitary adenomas. As such, accurate diagnosis often requires histopathological confirmation, with thyroid transcription factor-1 (TTF-1) emerging as a key immunohistochemical marker [2].

The management of PPTs is equally challenging. Surgical resection remains the cornerstone of treatment, with choice of approach (transsphenoidal vs. transcranial) dictated by tumor location and extension. However, outcomes vary significantly depending on tumor biology, surgical technique, and postoperative care [6, 8, 9, 12].

Endocrine and neurosurgical aspects of PPTs as well as the long-term outcome remain ill-defined. This retrospective study therefore addresses these gaps by evaluating the clinical and endocrine characteristics, surgical approaches, and survival outcome in a cohort of patients with histologically confirmed PPTs treated at our department.

## Methods

### Patient cohort

We identified all adult patients (≥ 18 years) who underwent surgery for pituitary lesions between 2000 and 2023 in our Department of Neurosurgery. From this database, we selected 19 patients with histologically confirmed diagnoses of PPTs (PCs, GCT, and SCOs). This study was conducted in compliance with the Declaration of Helsinki (1964) and its latest amendments.

### Data collection

Demographic characteristics, clinical presentations, endocrine status, imaging findings (tumor size, volume, and location), surgical approaches, histopathological reports, postoperative complications, conduction of radiation therapy, and follow-up data were retrospectively collected from patient medical records.

Endocrine evaluations consisted of assessments of morning fasting cortisol, adrenocorticotropic hormone (ACTH), thyroid-stimulating hormone (TSH), free thyroxine (fT4), follicle-stimulating hormone (FSH), luteinizing hormone (LH), testosterone (in males), estradiol (in females), growth hormone (GH), insulin-like growth factor-1 (IGF-1), and prolactin (PRL). Secondary adrenal insufficiency (AI) was defined as morning cortisol < 5 µg/dl and/or an insufficient cortisol response (< 18 µg/dl) to the 250 µg Synacthen stimulation test. All but one patient underwent Synacthen testing for confirmation. The one exception had repeatedly measured basal cortisol levels < 1 µg/dl, making additional dynamic testing unnecessary. Secondary hypothyroidism was diagnosed based on low fT4 with normal or low TSH. Hyperprolactinemia was defined as a serum prolactin level above the upper reference range. Hypogonadotropic hypogonadism was diagnosed in males with testosterone levels below the reference range and in premenopausal women with low estradiol levels or oligomenorrhea, amenorrhea, or infertility combined with low or normal gonadotropins. In postmenopausal women, hypogonadotropic hypogonadism was diagnosed by inappropriately low serum LH and/or FSH levels in relation to low estradiol level. GH deficiency was indicated by low IGF-1 levels relative to age- and sex-specific normative data. Due to the retrospective nature of the study, stimulation tests (e.g., insulin tolerance test, GHRH-arginine test) were not systematically performed, which may have led to an underestimation of GH deficiency prevalence. Arginine vasopressin deficiency (AVP-D) was diagnosed based on hypernatremia and/or serum hyperosmolality with hypotonic urine, polyuria, and polydipsia.

### Surgical approach and tumor resection

Gross total resection (GTR), defined as no visible residual tumor, was determined by comparing preoperative and postoperative magnetic resonance imaging (MRI), while subtotal resection (STR) was defined as incomplete tumor removal with visible tumor residue on postoperative MRI. Tumor volume was calculated using MRI measurements in three planes (axial, sagittal, and coronal) with the formula:$\:volume\:=\:(a\:\times\:\:b\:\times\:\:c)\:/\:2$

### Follow-up

Patients underwent comprehensive neurological, ophthalmological, and endocrinological evaluations preoperatively, at 7 days post-surgery, 3 months post-surgery, and annually thereafter. Follow-up MRI scans were performed at 3 months and annually thereafter. Recurrence was defined as MRI-confirmed tumor reappearance or progression irrespective of clinical symptoms.

### Neuropathological examinations

Surgical tissues were fixed in formalin, embedded into paraffin, cut into 3 μm thin sections and routinely stained for HE (haematoxylin and eosin) and PAS (periodic acid-Schiff) followed by histopathological examination. Further immunohistochemical stainings have been performed with an automated staining system (Ventana BenchMark ULTRA, Roche Diagnostics GmbH, USA) using following antibodies: TTF-1 (Clone 8G7G3/1; Cell Marque), S100 (Clone 4C4.9; Cell Marque), Vimentin (Clone V9; Dako), GFAP (Dako), EMA (Clone GP1.4; Leica), Ki-67(Clone MIB-1; Dako).

### Statistical analysis

Descriptive statistics were calculated for continuous variables as means and standard deviations (SDs), as all tested variables (age at surgery, tumor volume, BMI at different time points, and follow-up duration) followed a normal distribution according to the Shapiro-Wilk test (*p* > 0.05). Categorical variables were presented as numbers and percentages. For normally distributed continuous data, the Student’s t-test was used to compare independent groups, while the paired t-test was applied for dependent continuous data. Fisher’s exact test was used to compare proportions due to the small sample size. Overall survival was estimated using the Kaplan-Meier method, and patients who were lost to follow-up or died from non-tumor-related causes were censored at their last known follow-up date. All statistical analyses were conducted using SPSS Statistical Software (version 28.0, IBM Corp.), and statistical significance was defined as *p* < 0.05.

## Results

### Baseline demographics characteristics

We analyzed 19 patients with histologically confirmed PPTs: 3 PCs, 8 GCTs, and 8 SCOs (Table 1). Most tumors occurred in females (58%), with a female-to-male ratio of 1.38:1. GCTs and SCOs were more common in females (62.5% each, 1.67:1 ratio), while PCs were predominantly in males (66.7%, 2:1 ratio). The mean age at diagnosis was 57.2 ± 13.2 years: PCs at 66 ± 9.2 years, GCTs at 51.6 ± 15.1 years, and SCOs at 59.5 ± 11 years. Differences in age between subtypes or genders (male: 58.3 ± 13.5, female: 56.5 ± 13.6 years) were not statistically significant (*p* > 0.05).

### Clinical presentations

Of 19 patients, 15 (79%) were symptomatic, while 4 were incidentally diagnosed through imaging for unrelated conditions (Table 1). The most common symptoms leading to diagnosis were headache (31.6%), visual impairment (21%), and sexual dysfunction (10.5%). Adrenal crisis occurred in 3 patients, leading to hospitalization. Several patients exhibited multiple symptoms at the time of diagnosis. Overall, 7 patients (36.8%) experienced headaches, 7 reported visual impairments, and 7 had sexual dysfunction. Other notable symptoms included vertigo and fatigue, each reported in 4 patients. Symptoms varied by subtype: headaches predominated in GCTs (50%), visual disturbances in SCOs (50%), and adrenal crisis in PCs (66.6%). Sexual dysfunction was equally distributed across all subtypes.

**Table 1:** Demographic, clinical and endocrinological characteristics of patients with PPTs

	Overall *n* = 19	PCs *n* = 3	SCOs *n* = 8	GCTs *n* = 8
**Age (mean ± SD)**	57.2 ± 13.2	66 ± 9.2	59.5 ± 11	51.6 ± 15.1
**Gender**,** n (%)**				
Female	11 (58%)	1 (33.3%)	5 (62.5%)	5 (62.5%)
Male	8 (42%)	2 (66.7%)	3 (37.5%)	3 (37.5%)
**Symptom leading to diagnosis**				
- Headache	6 (31.6%)	0	2 (25%)	4 (50%)
- Visual impairment	4 (21%)	1 (33,3%)	2 (25%)	1 (12.5%)
- Adrenal crisis	3 (15.8%)	2 (66.7%)	1 (12.5%)	0
- Sexual dysfunction	2 (10.5%)	0	1 (12.5%)	1 (12.5%)
Incidental finding	4 (21%)	0	2 (25%)	2 (25%)
**Endocrine assessment**				
Hyperprolactinemia	6 (31,6%)	0	4 (50%)	2 (25%)
Hypothyroidism	12 (63%) (8*)	2* (66.7%)	5 (62,5%) (3*)	5 (62,5%) (3*)
Hypogonadism	8 (42%)	2 (66.7%)	2 (25%)	4 (50%)
Hypocortisolism	7 (36,8%)	2 (66.7%)	3 (37,5%)	2 (25%)
GH deficiency	6 (31,6%)	1 (33,3%)	2 (25%)	3 (37,5%)
AVP-D	0	0	0	0
**BMI (mean ± SD)**	27.46 ± 6.73	22.12 ± 2.4	24.74 ± 4.24	34.49 ± 5.72
**Follow-up (months**,** mean ± SD)**	63.9 ± 45.1	82.3 ± 57.5	52 ± 35.8	68.7 ± 53.5

*under L-Thyroxin supplement

### Tumor characteristics

Tumor location varied between the PPT subtypes (Table 2). Most GCTs (87.5%) were suprasellar, while 75% of SCOs involved both intra- and suprasellar regions. Only one patient had an exclusively intrasellar tumor (a PC case). Among the other PC patients, one had a suprasellar tumor and one had both intra- and suprasellar involvement (33.3% each). Mean tumor volumes were 1.20 ± 1.78 cm³ for PCs, 2.17 ± 1.57 cm³ for GCTs, and 2.78 ± 2.25 cm³ for SCOs, with no significant differences between subgroups (*p* > 0.05).

**Table 2:** Tumor characteristics and surgical outcomes

	Overall *n* = 19	PCs *n* = 3	SCOs *n* = 8	GCTs *n* = 8
**Tumor volume (mean ± SD**,** cm³)**	2.27 ± 1.9	1.20 ± 1.78	2.78 ± 2.25	2.17 ± 1.57
**Tumor location**,** n (%)**				
Intrasellar	1 (5.3%)	1 (33.3%)	0	0
Suprasellar	10 (52.6%)	1 (33.3%)	2 (25%)	7 (87.5%)
Intrasellar + suprasellar	8 (42.1%)	1 (33.3%)	6 (75%)	1 (12.5%)
**Surgical approach**,** n (%)**				
Transsphenoidal	9 (47.4%)	2 (66.7%)	6 (75%)	1 (12.5%)
Transcranial	10 (52.6%)	1 (33.3%)	2 (25%)	7 (87.5%)
**Extent of resection**,** n (%)**				
Gross total resection	11 (58%)	1 (33.3%)	5 (62.5%)	5 (62.5%)
Subtotal resection	8 (42%)	2 (66.7%)	3 (37.5%)	3 (37.5%)
**Complications related to surgery**,** n (%)**				
Deterioration of vision	6 (31.6%)	0	1 (12.5%)	5 (62.5%)
AVP-D	4 (21%)	1 (33%)	1 (12.5%)	2 (25%)
Transient SIAD	3 (16%)	1 (33%)	2 (25%)	0
Meningitis	3 (16%)	0	1 (12.5%)	2 (25%)
Epilepsy	2 (10.5%)	0	1 (12.5%)	1 (12.5%)
Epidural empyema	1 (5%)	0	1 (12.5%)	0
Motor aphasia	1 (5%)	0	0	1 (12.5%)
**Recurrence/progression**	3 (16%)	1 (33.3%)	2 (25%)	0
**Radiotherapy**	3 (16%)	1 (33.3%)	2 (25%)	0

Immunohistochemical analysis showed 100% positivity for TTF-1 and S-100 protein in all tumors (Table 3). Vimentin was expressed in 89.5% of cases, with 100% positivity in PCs and SCOs and 75% in GCTs. GFAP positivity was 52.6%, highest in PCs (67%). Glial fibrillary acidic protein (GFAP) was positive in 52.6% of cases, with the highest positivity rate in PCs (67%). Epithelial membrane antigen (EMA) was positive in 63%, most frequent in SCOs (75%), followed by GCTs (62.5%) and PCs (33%). The MIB-1 (Ki-67) proliferation index was below 3% in 84% of cases, but 33% of PCs and 25% of SCOs had higher rates (> 3%).

**Table 3:** Immunohistochemical characteristics of PTTs

Marker	Overall (%), *n* = 19	PCs (%), *n* = 3	SCOs (%), *n* = 8	GCTs (%) (*n* = 8)
TTF-1	19 (100)	3 (100)	8 (100)	8 (100)
S-100 protein	19 (100)	3 (100)	8 (100)	8 (100)
Vimentin	17 (90)	3 (100)	8 (100)	6 (75)
GFAP	10 (53)	2 (67)	4 (50)	4 (50)
EMA	12 (63)	1 (33)	6 (75)	5 (63)
MIB-1/Ki-67				
- <3%	16 (84%)	2 (67%)	6 (75)	8 (100)
- >3%	3 (16%)	1 (33%)	2 (25)	-

Abbreviations: TTF-1, thyroid transcription factor-1; GFAP, glial fibrillary acidic protein; EMA, epithelial membrane antigen; MIB-1/Ki-67 proliferation index

### Preoperative endocrine function

At diagnosis, 7 patients exhibited normal pituitary function, 2 had complete and 9 partial anterior pituitary insufficiency (Table 1). The most common dysfunction was hypogonadism (42%), followed by hypocortisolism (36.8%) and GH deficiency and hyperprolactinemia in equal measure (31.6%). One patient had isolated hyperprolactinemia. Assessment of the thyrotropic axis was affected by long-term L-thyroxine therapy in 8 patients. Among the remaining patients, 4 were newly diagnosed with hypothyroidism. Hypocortisolism was highest in PCs (67%), followed by SCOs (37.5%) and GCTs (25%). Hypogonadism was highest in PCs (67%) and GCTs (50%), and low in SCOs (25%). GH deficiency was observed in 33% of PC patients and 37.5% of GCT patients, but was present in only 25% of SCO patients. Hyperprolactinemia occurred in 50% of SCOs, 25% of GCTs, and was absent in PCs. Notably, no patients were diagnosed with AVP-D.

### Endocrine function one week after surgery

**Fig. 1:**
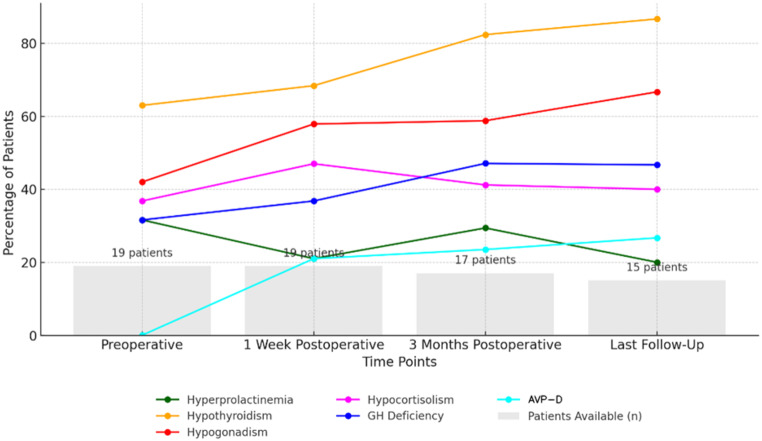
Endocrine dysfunction across time points in patients with PPTs. Trends in endocrine dysfunctions over four time points: preoperative, 1 week postoperative, 3 months postoperative, and last follow-up. Percentages calculated based on available cohort size

Postoperatively, hyperprolactinemia improved in all 6 preoperative cases, with 4 normalizing and 2 significantly reduced (Fig. 1). Two patients developed hyperprolactinemia, both with panhypopituitarism and AVP-D. AI was present postoperatively in 9 patients (47%), with 6 persistent and 3 new-onset cases. Only one patient had cortisol normalization. Hypogonadism worsened postoperatively, increasing to 57.9% from 42% preoperatively (Fig. 1). This was especially evident in SCOs, where hypogonadism increased from 25 to 62.5%. Thyroid function remained unchanged in thyroxine-treated patients, but two developed hypothyroidism, while one resolved. GH deficiency varied: one GCT patient improved, while new cases arose in one PC and one SCO patient. AVP-D developed in four patients (21%): two GCT, one PC, and one SCO case.

### Endocrine function 3 months after surgery

Seventeen patients were evaluated three months postoperatively. Hyperprolactinemia remained unchanged, except in one case associated with tumor progression (Fig. 1). AI persisted in 5 patients who had exhibited this condition preoperatively and immediately after surgery, normalized in 2, and developed newly in 2 others. Gonadal function remained impaired in 10 patients, with only one showing improvement after developing postoperative hypogonadism. Thyroid function remained unchanged in 13 patients on thyroxine therapy and one developed new hypothyroidism requiring treatment (Fig. 1). Accurate assessment was limited as thyroxine tapering or discontinuation was not performed. GH deficiency varied: one patient recovered but two were newly diagnosed. Overall, 8 patients had GH deficiency three months after surgery.

### Endocrine function at last follow-up

At the last follow-up, 15 patients were evaluated. Hyperprolactinemia remained unchanged except for one new-onset case linked to tumor progression and one normalization (Fig. 1). AI persisted in GCT patients and newly developed in one PC and one SCO patient. Gonadal function remained impaired in 10 patients, with one SCO male developing new-onset hypogonadism. Thyroid function improved in one patient from each tumor group, allowing discontinuation of thyroxine therapy. GH deficiency showed varied outcomes: one new case occurred each in PC and GCT patients, while two GCT patients recovered. Among the three patients with worsening pituitary function, two had tumor progression, while one had a stable tumor remnant. AVP-D persisted unchanged in all affected patients postoperatively.

### Surgical outcomes and complications

Of the 19 patients, 9 (47.3%) underwent transsphenoidal surgery (TSS), and 10 (52.6%) underwent open transcranial surgery (OTS) (Table 2). Most GCT patients (87.5%) had OTS, while 75% of SCO patients and 67% of PC patients underwent TSS. Tumor location influenced the approach, with intrasellar or combined tumors favoring TSS and suprasellar tumors requiring OTS. GTR was achieved in 11 patients (58%), while 8 (42%) underwent STR. GTR rates were similar in GCT and SCO groups (62.5%). STR decisions were influenced by increased tumor vascularity, more firm consistency, and surrounding structure infiltration. The pituitary stalk was anatomically preserved in most cases; however, intraoperative assessment revealed varying degrees of compression and displacement due to the tumor mass effect. In several cases, the stalk appeared significantly thinned or adherent to the tumor, while in others, partial disruption occurred due to tumor infiltration or firm adherence; however, a complete stalk resection was not performed in any case. The most common postoperative complications (Table 2) were deterioration of vision (31.6%), AVP-D (21%), transient SIAD and meningitis (16% each), epilepsy (10.5%), and rare cases of epidural empyema and motor aphasia (5% each). OTS had a higher risk of visual impairment (60%) compared to TSS (0%). Postoperative AVP-D rates were similar: 22% in the TSS group and 20% in the OTS group.

### Body mass index (BMI) analysis

**Fig. 2:**
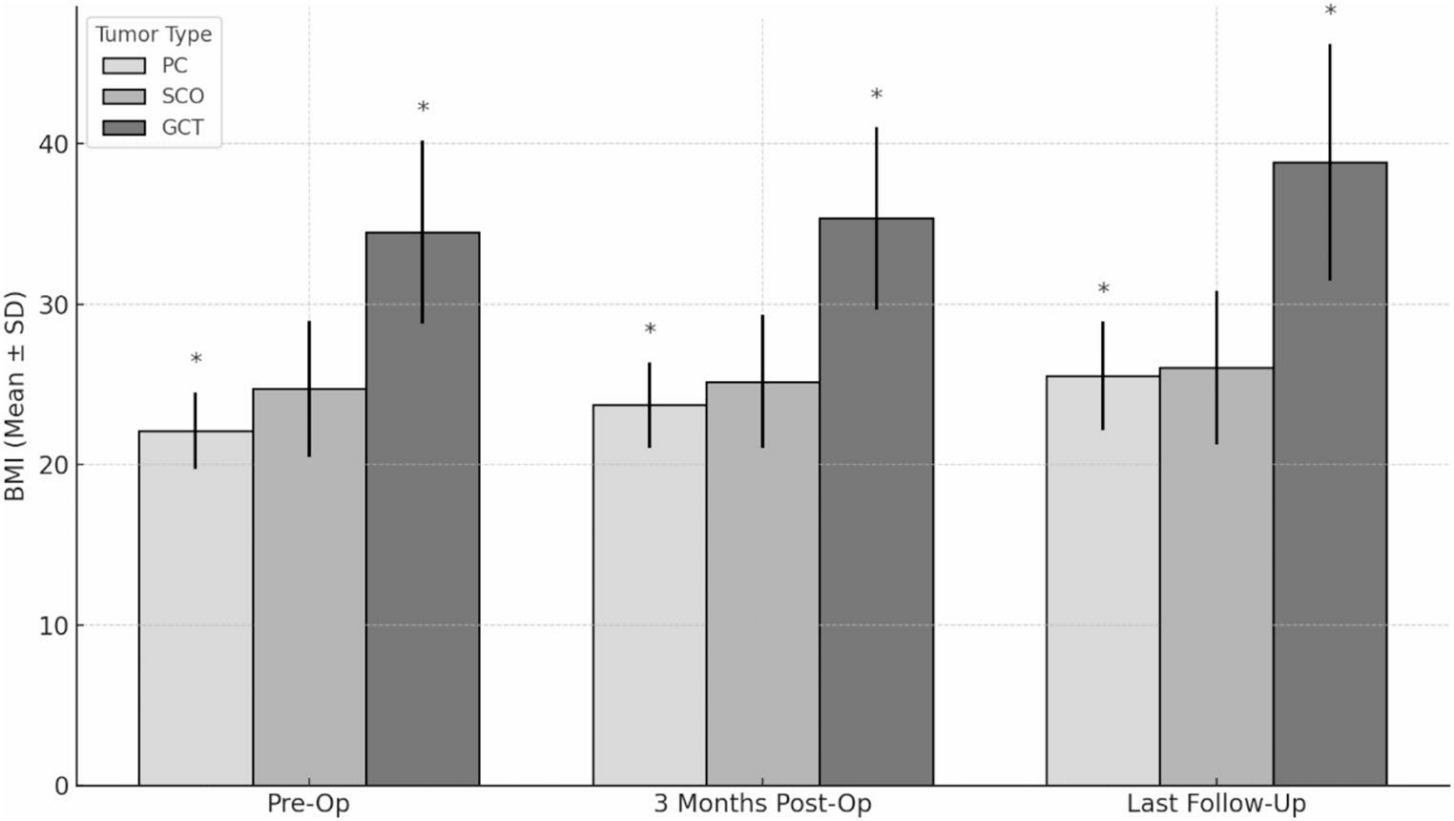
BMI changes by tumor type over time. The graph illustrates the changes in BMI for patients with PPTs, categorized by tumor type. BMI was assessed preoperatively (Pre-Op), at 3 months postoperatively (3 Months Post-Op), and at the latest follow-up (Last Follow-Up). Statistically significant differences in BMI are marked with an asterisk (*), indicating a p-value < 0.05

BMI was assessed pre-surgery, 3 months post-surgery, and at the latest follow-up. The mean pre-operative BMI was 27.46 ± 6.73, which increased significantly to 28.28 ± 6.69 three months after surgery (*p* = 0.002) (Table 1; Fig. 2). At the latest follow-up, the mean BMI further increased to 30.21 ± 8.19 (*p* < 0.001), indicating a statistically significant long-term weight gain. The observed changes in BMI therefore reflect a short-term weight increase following surgical intervention and suggest a continued trend of long-term weight gain in patients with PPTs. When analyzing BMI according to tumor type, patients with GCT had a significantly higher pre-operative mean BMI (34.49 ± 5.72) compared to those with PC (22.12 ± 2.40) and SCO (24.74 ± 4.24), (*p* < 0.01). At 3 months and at the latest follow-up, GCT patients continued to have significantly higher BMIs compared to the other groups (*p* < 0.01). Intergroup differences remaining statistically significant over time suggest that while surgery contributes to overall weight gain, disparities between tumor types persist. All patients requiring glucocorticoid replacement received physiological hydrocortisone doses (15–25 mg/day); none received supraphysiological or high-dose steroids. No significant correlation was found between steroid use and BMI increase (*p* = 0.57–0.23, *r* = -0.17 to -0.34). Imaging showed hypothalamic contact or invasion in some cases, but no significant correlation with BMI changes (Spearman ρ = -0.12 to -0.24, *p* > 0.3) or tumor location (intrasellar vs. suprasellar, ρ = 0.09–0.15, *p* > 0.5).

### Follow-up and outcomes

**Fig. 3:**
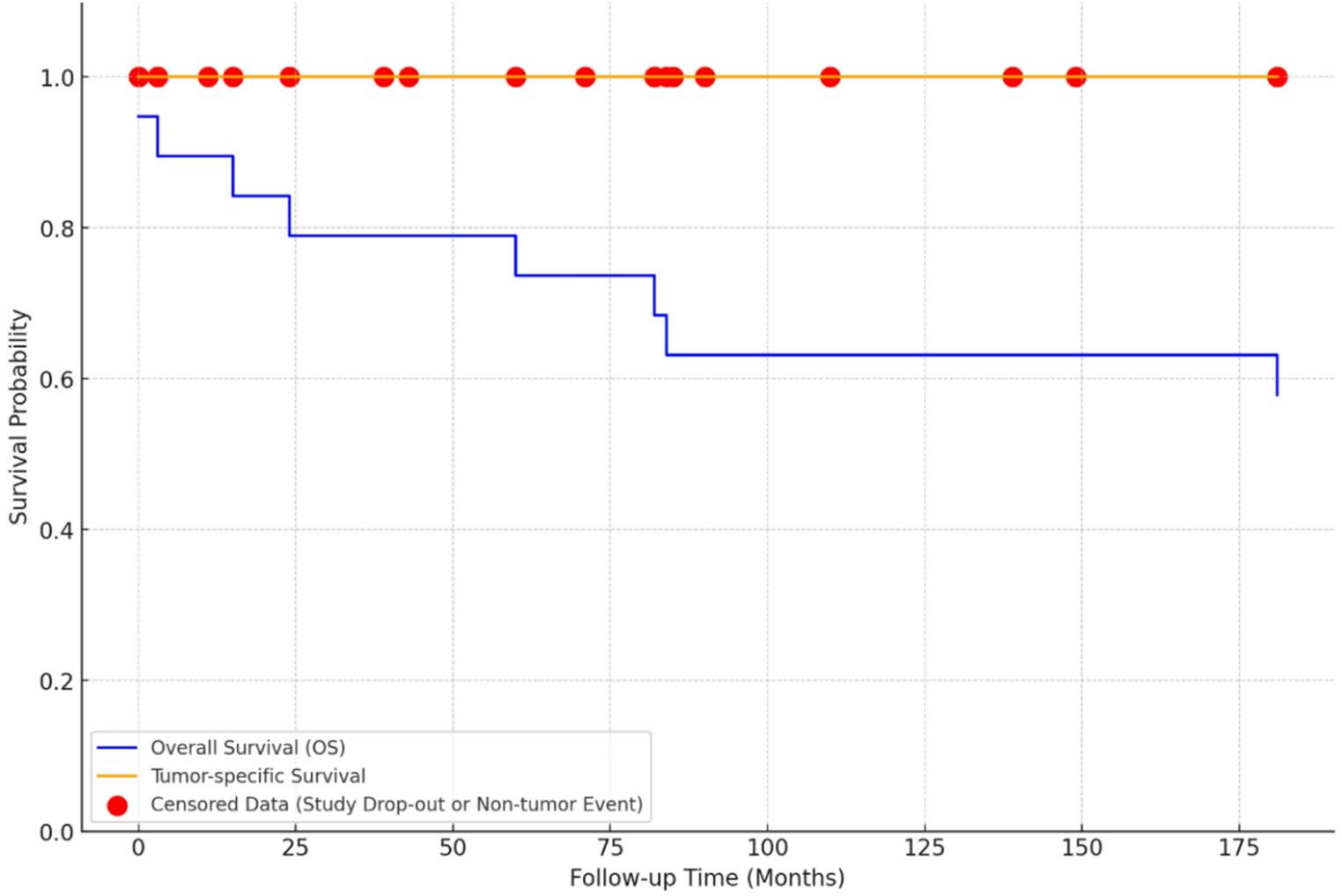
Kaplan-Meier survival curve: overall survival vs. tumor-specific survival

The mean follow-up period was 63.9 ± 45.1 months, with the longest follow-up in the PC group (82.3 ± 57.5 months) (Table 1). 3 patients (16%) experienced tumor recurrence, including one from the PC group and two from the SCO group. These patients underwent radiation therapy for tumor progression (Table 2). Seven patients died during the follow-up period. None of the mortalities were tumor-related. The causes of death were varied and included cardiopulmonary arrest on the fourth postoperative day (1 patient), necrotizing pancreatitis after 60 months of follow-up (1 patient), and newly diagnosed colorectal cancer at 87 months postoperatively (1 patient). Three patients died from cardiac disease at 17, 87, and 181 months, and one from type 1 diabetes complications at 23 months. Overall survival rates were 88% at 3 years and 79.8% at 5 years, reflecting the cohort’s advanced age and non-tumor-related comorbidities despite the generally favorable prognosis (Fig. 3).

## Discussion

PPTs are rare non-neuroendocrine neoplasms that pose diagnostic and management challenges due to their rarity, overlapping clinical features, and radiological similarities to other sellar lesions [13]. Despite their low incidence, they are clinically significant for their potential to cause long-term neurological and endocrine dysfunctions [9].

Our single-center study, comprising 19 patients with histologically confirmed PPTs, provides insights into their presentation, management, and outcomes. The demographic characteristics of our cohort align with previous studies. Consistent with Covington et al. and other meta-analyses [6, 14, 15], we found a female predominance for GCTs and SCOs, while PCs were more common in males. The mean age at diagnosis for GCTs (51.6 years) and SCOs (59.5 years) aligns with prior reports [7, 11], while PCs (66 years) were diagnosed later than in other series [14], possibly reflecting delayed presentation.

PPTs grow slowly, with symptoms primarily resulting from mass effect or compression of surrounding structures. Their clinical presentation is nonspecific and resembles other sellar lesions [16, 17]. In our series, headache (31.6%) and visual impairment (21%) were the most frequent symptoms, consistent with findings by Chen et al. and others [11–14], followed by sexual dysfunction and symptoms of AI. Symptoms varied among PPT subgroups, with headache more common in GCTs [7, 18] and visual disturbances more frequently observed in PCs and SCOs [8, 12, 19]. Other symptoms, including dizziness, decreased libido, nerve paralysis, fatigue, and nausea, occurred at rates comparable to those reported in the literature. A notable finding in our study was the significant subset of patients presenting with multiple simultaneous symptoms, a phenomenon underrepresented in previous reports. Additionally, three patients initially presented with adrenal crisis, underscoring the potential for PCs to cause acute AI through pituitary stalk compression and disruption of the hypothalamic-pituitary-adrenal axis [9].

Our study underscores the metabolic effects of PPTs, demonstrating progressive BMI increases across all subtypes (Fig. 2), with GCT patients exhibiting the highest preoperative BMI and further postoperative gain. While prior studies suggest hypothalamic involvement in GCTs may disrupt energy regulation and contribute to weight gain [2], our analysis found no significant correlation with BMI changes (Spearman ρ = -0.12 to -0.24, *p* > 0.3), indicating that additional factors are likely involved. However, other signs of hypothalamic dysfunction, such as hyperphagia, autonomic dysregulation, or neurocognitive symptoms, were not systematically documented, limiting definitive conclusions. Recent studies reveal distinct methylation profiles and mutation spectra in GCTs compared to PCs and SCOs, emphasizing tumor biology’s role in metabolic changes [5].

GCTs in our cohort were predominantly suprasellar, consistent with Zhang et al.‘s report of 57.7% in this region [7]. In contrast, pooled analyses of 141 cases found 35.5% suprasellar and 28.4% combined suprasellar-intrasellar locations [20]. SCOs were commonly observed in both intra- and suprasellar regions, aligning with previous studies [8, 9, 14]. Purely intrasellar PPTs were rare, in agreement with prior reports. Notably, PCs were the only tumors confined to the intrasellar region in our cohort, reflecting their known anatomical distribution.

The mean tumor volumes in our cohort were smaller than those reported in other studies with smaller cohorts. A review reported a mean volume of 4.73 cm³ for PCs [21], while a large case series by Qiao et al. noted median volumes of 2.1 cm³ overall, 2.6 cm³ for SCOs, and 1.7 cm³ for PCs and GCTs [1], aligning with our findings. Vuong et al., in contrast, reported a larger mean volume of 19.3 cm³ for SCOs, with a broad range (1.8–148.5 cm³), demonstrating substantial variability [10].

Immunohistochemical analysis confirmed TTF-1 positivity in all tumors, consistent with the WHO classification, where TTF-1 is a diagnostic hallmark of PPTs [2, 3, 18]. S-100 protein positivity matched previous reports [2]. However, staining patterns for vimentin, GFAP, and EMA varied, indicating heterogeneity among PPT subtypes. Our findings align with Chen et al. for PCs [22] and Zhang et al. for GCTs [7], though GFAP positivity in SCOs (50%) exceeded the 10.8% reported by Giantini et al. [23]. Most PPTs in our study had a low MIB-1 (Ki-67) index (< 3%), consistent with prior findings [1, 18, 21]. However, 33% of PCs and 25% of SCOs showed higher rates (> 3%), indicating variability in aggressiveness and highlighting the need for tailored monitoring and treatment approaches.

Endocrine dysfunctions in PPT patients emphasize the chronic and diverse hormonal disruptions caused by these tumors. AI affected over a third of patients preoperatively and persisted or worsened in nearly half postoperatively. Hypogonadism was common, affecting nearly half preoperatively and increasing post-surgery, reflecting gonadotropic cell vulnerability. In contrast, hyperprolactinemia improved or resolved postoperatively, likely due to relief of tumor-induced pituitary stalk compression. GH deficiency varied, with both recovery and new-onset cases during follow-up. However, as our study primarily relied on IGF-1 levels as a surrogate marker for GH deficiency, without systematic stimulation testing, the true prevalence of GH deficiency may be underestimated, especially in older adults. Although preoperative AVP-D has been reported in other series, it was not observed in our cohort. This may be attributed to differences in tumor distribution or the extent of hypothalamic or pituitary stalk involvement. Postoperatively, 21% developed AVP-D, suggesting an iatrogenic rather than primary origin, consistent with Roncaroli et al.‘s findings on the posterior pituitary’s surgical susceptibility [15]. Endocrine deficits post-surgery suggest that resection rarely restores full pituitary function, consistent with reports of irreversible hypothalamic-pituitary axis damage in PPTs [1, 7, 9, 14, 21, 24]. Furthermore, the occurrence of persistent or new-onset endocrine dysfunctions in our cohort underscores the need for comprehensive endocrine follow-up, as supported by studies reporting similar dysfunction rates [6–9, 14, 21, 25].

GTR remains the standard in oncology. Surgical approach depends on tumor location, extension, and proximity to critical structures like the optic chiasm, hypothalamus, and pituitary stalk [6–8, 12, 14]. TSS is preferred for intrasellar tumors, while OTS is suitable for significant suprasellar or parasellar extension. In our cohort, 48% underwent TSS and 52% OTS. Guerrero-Perez et al. reported TSS as the most common technique (64.4%, 132/205) in published PPT cases [9]. GCTs were predominantly treated with OTS due to their suprasellar location, while SCOs were frequently resected via TSS, consistent with Zhang et al. [7]. Our GTR rate of 58% falls within reported ranges (50–60%) [1, 9, 10]. Complete resection is often hindered by firm adhesion to surrounding structures and rich vascularity, challenges also highlighted in reviews of surgical outcomes for PCs and SCOs.

Postoperative complications in our series align with previous reports [6, 8, 9, 12, 14, 15, 18]. AVP-D incidence did not significantly differ between surgical approaches (TSS: 2/9; OTS: 2/10), suggesting approach choice has little impact on AVP-D risk.

In contrast, OTS was associated with significantly more complications, affecting all 10 patients, compared to 4 of 9 in the TSS group. These findings support prior studies indicating OTS carries a higher risk of optic system damage, while TSS increases subtotal resection likelihood [26]. Although AVP-D risk is comparable between approaches, OTS presents a greater overall complication burden, underscoring the importance of preoperative risk stratification, particularly in lesions amenable to TSS.

Studying PPTs remains challenging due to their rarity, with existing case series often limited by small size and heterogeneity, introducing selection bias in pooled analyses [21, 22, 26–29]. In our cohort (mean follow-up: five years; longer for PCs, shorter for SCOs), tumor recurrence or progression occurred in three patients (1 PC, 2 SCO; 15.8%), all requiring adjuvant radiation therapy. This recurrence rate is lower than the 35% reported in some meta-analyses, likely reflecting differences in surgical techniques, patient selection, or follow-up duration [6–14]. Similarly, our radiation therapy rate (15.8%) was lower than the 26% reported for residual tumors [9].

Kaplan-Meier analysis showed excellent 5-year survival in PPT patients (Fig. 3), consistent with previous studies [27, 29]. Tumor-specific survival was 100%, with no tumor-related deaths, while overall survival was approximately 80%, with deaths attributed to non-tumor causes. The high number of censored data points suggests that mortality was influenced by other health factors rather than the tumor. These findings underscore the indolent nature of PPTs and their favorable prognosis, especially with appropriate surgery and long-term follow-up.

## Conclusion

PPTs are rare tumors with overlapping clinical and radiological features, making diagnosis challenging. Histopathological examination and immunohistochemical markers, especially TTF-1, are crucial for accurate classification. Despite their indolent nature and favorable 5-year tumor-specific survival (100% in our cohort), PPTs cause significant morbidity. Persistent endocrine dysfunctions, including hypocortisolism, hypogonadism, and GH deficiency, are common pre- and post-surgery. These findings underscore the importance of early endocrine evaluation and long-term management to enhance patient quality of life.

The surgical approach for PPTs is critical, with transsphenoidal surgery linked to fewer complications but limited resection in suprasellar or parasellar cases. Transcranial approaches carry higher risks, including visual impairment. Significant postoperative weight gain highlights the need for metabolic monitoring and intervention to mitigate long-term risks such as metabolic syndrome and cardiovascular disease.

In summary, PPTs require a multidisciplinary approach encompassing individualized surgical planning, precise histological diagnosis, and proactive management of endocrine and metabolic complications to optimize patient outcomes.

## Data Availability

The data supporting the findings of this study are not publicly available due to patient confidentiality but can be made available upon reasonable request from the corresponding author.
